# Advanced Zinc Anode with Nitrogen‐Doping Interface Induced by Plasma Surface Treatment

**DOI:** 10.1002/advs.202103952

**Published:** 2021-11-26

**Authors:** Hao Jia, Minghui Qiu, Chuntao Lan, Hongqi Liu, Mahmut Dirican, Shaohai Fu, Xiangwu Zhang

**Affiliations:** ^1^ Key Laboratory of Eco‐Textiles Ministry of Education Jiangnan University Wuxi Jiangsu P.R. China; ^2^ Fiber and Polymer Science Program Department of Textile Engineering Chemistry and Science Wilson College of Textiles North Carolina State University Raleigh NC USA

**Keywords:** N‐doped interfaces, Plasma surface treatment, Zn anodes, Zn‐ion batteries

## Abstract

Aqueous zinc‐ion batteries (ZIBs) are one of the most ideal candidates for grid‐scale energy storage applications due to their excellent price and safety advantages. However, formation of Zn dendrites and continuous side reactions during cycling result in serious instability problems for ZIBs. In this work, the authors develop a facile and versatile plasma‐induced nitrogen‐doped Zn (N‐Zn) foil for dendrite‐free Zn metal anode. Benefitting from the uniform nucleation sites and enhanced surface kinetics, the N‐Zn anode exhibits exceptionally low overpotential (around 23 mV) at 1 mA cm^−2^ and can be cycled for over 3000 h under 1 mA cm^−2^ because of the enhanced interface behavior. The potential application of N‐Zn anode is also confirmed by introducing a full Zn/MnO_2_ battery with outstanding capacity stability for 2000 cycles at 1 A g^–1^. Overall, this work offers new fundamental insights into homogenizing Zn electrodeposition processes by pre‐introduced active nucleation sites and provides a novel direction of interface design engineering for ultra‐stable Zn metal anode.

## Graphical abstract

A plasma technique is introduced to form an N‐doped interface on the Zn metal anode for high stability ZIBs. The plasma‐induced N‐doped Zn electrode can form uniform active sites for guiding a homogenized electrodeposition process and effectively reduce the Zn ion diffusion barrier. The N‐Zn electrode exhibits exceptionally low overpotential and ultra‐long lifespan due to the enhanced interface behavior.

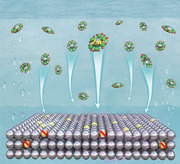

## 1 Introduction

Increasing energy demand in recent years has prompted the development of advanced energy storage devices. Although lithium‐ion batteries (LIBs) remain the dominant energy storage system, a wide variety of batteries and other electrochemical products will coexist with their intrinsic characteristics in the future.^[^ ^1^, ^2^ ^]^ In this regard, some price‐friendly aqueous metal‐ion batteries, such as zinc‐ion batteries (ZIBs), are particularly suitable for grid‐scale energy storage applications.^[^ ^3^, ^4^ ^]^ Aqueous ZIBs are an emerging energy storage technology with low cost, large capacity and high safety.^[^ ^5^, ^6^ ^]^ Compared with LIBs using flammable and toxic organic electrolytes, aqueous ZIBs have great commercial potential in large‐scale energy storage systems and high‐safety wearable devices, which require close contact with the human body.

For a long time, most research has focused on the fabrication of novel cathode materials; however, the original zinc (Zn) foil is still being used as the anode material in ZIBs without any modification.^[^ ^7^ ^]^ Traditional Zn anode has several disadvantages, such as dendrite growth, self‐corrosion, and other ancillary side reactions. As a result, the overall cycle stability and safety performance of ZIBs are far from meeting practical application requirements.^[^ ^8^ ^]^ Notably, Zn dendrites are formed on Zn foil due to the uneven electric field distribution on the anode surface during the repeated plating/stripping process. The presence of Zn dendrites aggravates the parasitic reaction of the electrolyte, produces more by‐products, increases the battery impedance and even penetrates into the separator, resulting in a rapid capacity fade and even short circuits inside the battery.^[^ ^9^ ^]^

Many efforts have been devoted to improving the electrochemical performance of the Zn anode, such as novel anode structure design and electrolyte composition optimization.^[^ ^10^ ^]^ Among them, reconstructing an elaborate artificial interface on the Zn anode surface is widely regarded as a meaningful strategy to restrain Zn dendrite formation and prevent side reactions because of the enhanced interfacial behavior.^[^ ^11^, ^12^, ^13^, ^14^ ^]^ For instance, introducing an insulating coating layer (CaCO_3_, TiO_2_ and Al_2_O_3_) could effectively adjust external Zn^2+^ flux and further depress the Zn dendrite growth owing to their high porosity and excellent corrosion resistance.^[^ ^15^, ^16^, ^17^, ^18^ ^]^ However, Zn dendrites still inevitably grow during the plating process and eventually cause damage to the passivation layer structure. Although conductive material coatings, such as carbon nanotubes or Mxenes, could ameliorate the interior electric field and reduce interface impedance, Zn nuclei formed on these conductive surfaces could still cause Zn dendrite formation during repeated plating processes.^[^ ^19^, ^20^ ^]^ Hence, it is still highly desirable to explore a high‐efficient modification method on crude Zn anode for commercial application. Actually, bare Zn metal reacts with oxygen to generate a passivation layer of zinc oxide in the natural environment and normally presents poor electrolyte wettability.^[^ ^21^ ^]^ Therefore, it is essential to enhance zincophilicity of the Zn surface by introducing some exotic chemical structures such as free radicals and active groups that will improve the Zn plating performance.

Herein, we introduced a plasma technique, for the first time, to form a nitrogen (N)‐doped interface on a Zn metal anode (denoted as N‐Zn) for high stability ZIBs. The N_2_ plasma can effectively produce deep nitrogen doping in bare Zn metal via the high‐energy‐ionized nitrogen gas bombardment. The plasma‐induced N‐doped Zn electrode can form uniform active sites for guiding a homogenized electrodeposition process on account of the strong binding force between Zn^2+^ and N. The diffusion barrier of Zn^2+^ migration on the N‐doped electrode and charge‐transfer resistance could also be effectively reduced. Thanks to the enhanced surface characteristic, the symmetric cell of the N‐Zn electrode achieved an ultra‐stable lifespan of 3000 h under 1 mAh cm^–2^. Moreover, the advantage of the N‐Zn anode was further verified by a Zn/MnO_2_ full battery that exhibited ultra‐stable cycling behavior for 2000 cycles at 1 A g^–1^.

## 2 Results and Discussion

A relatively long exposure time to plasma treatment is considered as an effective approach to induce the functionalization on the Zn metal surface because of the prolonged contact between the active particles in the plasma and the Zn metal surface.^[^ ^22^ ^]^ However, the functionalization process cannot be boosted when the treatment time exceeds a particular threshold. Instead, there will be a decrease in the number of active groups resulting from the destruction of the existing active groups by the newly created active particles. According to the preliminary experiment, the commercial Zn foil was treated by nitrogen gas plasma (200 W) for an optimal time of 2 mins at room temperature to obtain the N‐Zn foil with the largest N‐doping content as shown in Table S1, Supporting Information.

**Figure 1:**
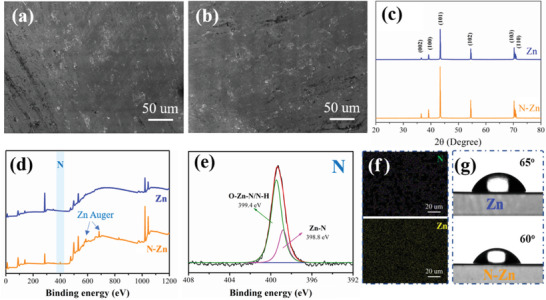
**F**SEM images of a) bare Zn and b) N‐Zn foils. c) XRD and d) XPS patterns of bare Zn and N‐Zn foils. e) High‐resolution XPS N 1s spectrum of N‐Zn foil. f) EDS mapping of N‐Zn foil. g) Contact angles of the 2 m ZnSO_4_ electrolyte on bare Zn and N‐Zn foils after standing for 20 min.

The morphology of the obtained N‐Zn foil was first investigated by a field emission scanning electron microscope (FSEM), while the untreated bare Zn foil was used as a control sample (**Figure** **1**a,b). It is notable the surface of the N‐Zn foil presented no significant change after the treatment of high energy nitrogen gas due to the strong hardness of Zn metal.^[^ ^23^ ^]^ As the initial electric field distribution plays a key role in the Zn nucleation process, the poor flatness of the Zn surface generally causes uneven electrodeposition as a result of the inhomogeneous electric field distribution.

The X‐ray diffraction (XRD) peaks of N‐Zn did not change in comparison to the bare Zn indicating the limited amount of N‐doping (Figure 1c). Moreover, the wide scan X‐ray photoelectron spectrum (XPS) of N‐Zn displayed in Figure 1d demonstrated the presence of N 1s peak at 398.9 eV with a surface atom percentage of 8%. Moreover, the attack of nitrogen plasma is conducive to removing pollutants on the Zn surface to a certain extent. Thus, some peaks of Zn auger were increased as the signal of Zn improved. Figure 1e also manifested that N 1s peaks could be obtained for both O‐Zn‐N/N‐H (399.4 eV) and Zn‐N (398.8 eV) in the high‐resolution XPS N 1s spectrum of N‐Zn.^[^ ^24^ ^]^ Additionally, the energy‐dispersive spectroscopy (EDS) mapping clearly detected the N element on the N‐Zn surface, indicating that the N heteroatoms were uniformly doped onto the Zn foil after N_2_ plasma treatment (Figure 1f), and accounts for about 6% of the total mass (Figure S1, Supporting Information). The electron probe X‐ray microanalyzer (EPMA) also further confirmed the uniform nitrogen distribution on the treated Zn surface (Figure S2, Supporting Information). More impressively, as presented in Figure 1g and Figure S3, Supporting Information, though the surface wettability (the contact angle) of the N‐Zn foil with 2M ZnSO_4_ electrolyte (97°) was slightly enhanced compared to that of Zn foil (98°), the electrolyte affinity difference between these two electrodes increased significantly over time. After a period of 20 min, the contact angles were tested as 65° and 60° for Zn and N‐Zn foils, respectively. As the stronger electrolyte affinity implies the lower interfacial free energy between the electrode substrate and the electrolyte, it demonstrates that the introduced nitrogen‐containing active group on the N‐Zn surface favors the relatively uniform electrolyte flux towards the electrode surface.^[^ ^25^ ^]^ The effect of the N‐doped surface layer on the Zn corrosion was also detected by the linear polarization experiments in an electrolyte of 2 m ZnSO_4_ (Figure S4, Supporting Information). The corrosion potential of the N‐Zn increased from −0.9964 to −0.9938 V when compared to the bare Zn, indicating a higher electrochemical stability of the N‐Zn electrode against the corrosion in the electrolyte. Also, the improved corrosion potential of the N‐Zn electrode suggested it had a lower risk of corrosion reactions than the bare Zn electrode.^[^ ^26^ ^]^

**Figure 2:**
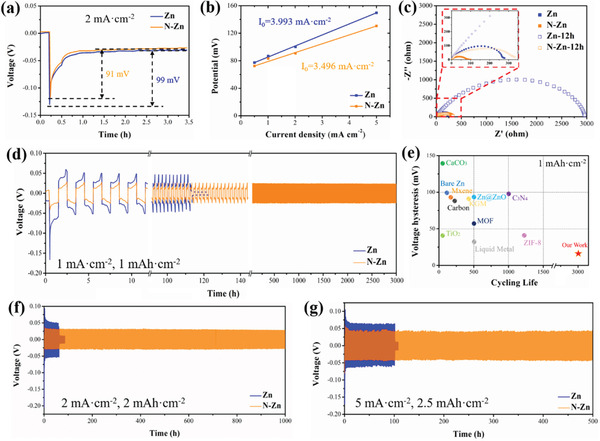
a) Nucleation overpotential of bare Zn and N‐Zn electrodes (vs. Zn electrode) at 2 mA cm^–2^ and b) corresponding exchange current density from the fitted curves under different scan rates tested at room temperature. c) Nyquist plots of bare Zn and N‐Zn symmetrical cells at the initial state and after standing for 12 h. d) Long‐term cycling performance of bare Zn and N‐Zn symmetrical cells at a current density of 1 mA cm^–2^ for 1 mAh cm^–2^. e) The comparison of voltage hysteresis and lifespan of N‐Zn with those of other literature reported surface‐modified anodes. Short‐term cycling results of bare Zn and N‐Zn symmetrical cells at current densities of f) 2 mA cm^–2^ for 2 mAh cm^–2^ and g) 5 mA cm^–2^ for 2.5 mAh cm^–2^.

To understand the function of the N‐doping interface in reducing Zn deposition barriers, nucleation overpotential of bare Zn and N‐Zn foil was measured against Zn at 2 mA cm^–2^. It is manifested from **Figure** **2**a that N‐Zn displayed a nucleation overpotential of 91 mV, which was lower than that of bare Zn (99 mV). Similarly, the reduced nucleation overpotentials of the N‐Zn electrode were also demonstrated under 0.5, 1 and 5 mA cm^–2,^ as illustrated in Figure 2b. Moreover, the exchange current density associated with the Zn electrodeposition process was calculated to evaluate the kinetics of deposition using the Butler‐Volmer approximation equation (1)

(1) $$i=i_{0}\frac{F}{RT}\cdot \frac{\eta }{2}$$

where *F* is the Faraday's constant, *R* is the ideal gas constant, *T* is the Kelvin temperature, and *η* is the overpotential of reduction.^[^ ^27^, ^28^ ^]^ As shown in Figure 2b, N‐Zn delivered a lower exchange current density (3.496 mA cm^–2^) than bare Zn (3.993 mA cm^–2^), implying a smaller critical radius of nucleation and faster redox reaction rate in the initial electrocrystallization period.^[^ ^29^ ^]^

For the electrochemical durability of Zn and N‐Zn electrodes during galvanostatic cycling, symmetrical coin cells with two identical bare Zn and N‐Zn foils were assembled with a concentrated aqueous electrolyte of 2 m ZnSO_4_. The electrical impedance spectrum (EIS) characterizations for Zn and N‐Zn symmetry cells were first conducted as displayed in Figure 2c. It is obvious that the charge transfer resistance (*R_ct_*) of Zn cells could be effectively reduced from 270 Ω to around 70 Ω by the surface N‐doping treatment. Hence, the N‐doping strategy can significantly enhance the Zn metal surface in both reactivity and electrical conductivity. This finding suggests that accelerated charge transfer on the N‐Zn surface easily formed dense charge density, which could induce uniform Zn deposition. More impressively, the *R_ct_* of N‐Zn cells remained at 320 Ω after standing for 12 h in contrast to that of Zn cells as over 2800 Ω, indicting a greatly boosted anticorrosion performance of N‐doped surface. Additionally, as shown in Figure 2d, at a current density of 1 mA cm^–2^ and capacity of 1 mAh cm^–2^, the symmetrical N‐Zn cell delivered stable voltage plateaus for over 3000 h without any short‐circuit. Notably, the voltage hysteresis of the N‐Zn cell was only around 23 mV. In contrast, the cells with bare Zn electrodes could cycle for no more than 150 h with an enlarged voltage hysteresis (around 50 mV) due to the growth of Zn dendrites as well as the byproduct formation. Figure 2e summarizes the voltage hysteresis and lifespan of N‐Zn along with those of other related works introducing artificial surface modification methods for the Zn anode of ZIBs.^[^ ^15^, ^16^, ^27^, ^30^, ^31^, ^32^, ^33^, ^34^, ^35^ ^]^ It is worth noting that this work shows loner cycling life and lower voltage hysteresis under 1mA cm^–2^ compared to other reported approaches. In addition, the symmetrical N‐Zn cell maintained a stable lifespan for 1000 h at 2 mA cm^−2^ and 500 h at 5 mA cm^–2^, demonstrating the high reversibility of zincophilic sites under large current density.

**Figure 3:**
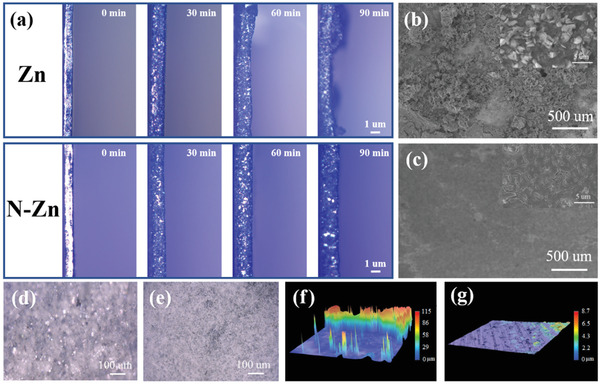
a) In situ optical microscopy visualization of Zn plating on bare Zn and N‐Zn at 5 mA cm^–2^. FSEM images of b) bare Zn electrode after 50 cycles and c) N‐Zn electrode after 500 cycles. The optical and three‐dimensional depth distribution images of the above cycled d, f) Zn and e, g) N‐Zn electrodes in the symmetrical cells.

The macroscopic morphological evolution of the electrode/electrolyte interface at different stages during the Zn plating process was in situ visualized by optical microscopy using a homemade imaging device. As displayed in **Figure** **3**a, the surface of the bare Zn electrode exhibited some fine protuberances after 30 min of plating. The as‐formed protrusions further exacerbated the abnormal distribution of the electrical field and ion flux at the interface, finally evolving into serious Zn dendrites after 90 min. In comparison, the N‐Zn electrode maintained a smooth surface and no visible dendrites were observed during the whole plating process, indicating that the active sites of electronegative N atoms could play a key role in achieving uniform Zn nucleation and deposition. The evenly dispersed N could strongly appeal to the initial Zn nucleation and uniform the subsequent growth of Zn nuclei, finally ensuring a dense packing of the Zn deposition.^[^ ^36^ ^]^ In addition, surface morphologies of the Zn and N‐Zn electrodes after plating cycles were studied by ex situ FSEM measurements (Figure 3b,c). The inhomogeneous mossy‐like Zn deposition was clearly observed on the Zn surface while there was no obvious morphological change for the N‐Zn electrode, which indicated that the preliminary N‐doping sites contributed to form uniform nucleation sites and reduced dendrite growth during Zn plating process. Apart from this, the frontal optical visualization observation demonstrated that the bare Zn electrode suffered stability attenuation upon 50 cycles due to dendrite growth while the N‐Zn electrode could achieve a homogeneous Zn deposition for 500 cycles (Figure 3d and e). The strong XRD reflection peaks of zinc hydroxide sulfate (Zn_4_SO_4_(OH)_6_·5H_2_O) were detected for the cycled Zn electrode in Figure S5, Supporting Information, but no byproduct was notably detected for the cycled N‐Zn electrode owing to the depressed corrosion rate. In addition, the severe swelling problem caused by the dendritic growth and side reactions of the Zn electrode was alleviated for the N‐Zn electrode, as demonstrated in Figure S6, Supporting Information. Moreover, corresponding three‐dimensional depth distribution images (Figure 3f,g) validated the surface maintenance function of the uniform N‐doping on avoiding the tip‐effect‐induced Zn dendrite growth, as revealed by the decreased exchange current densities as demonstrated before.

**Figure 4:**
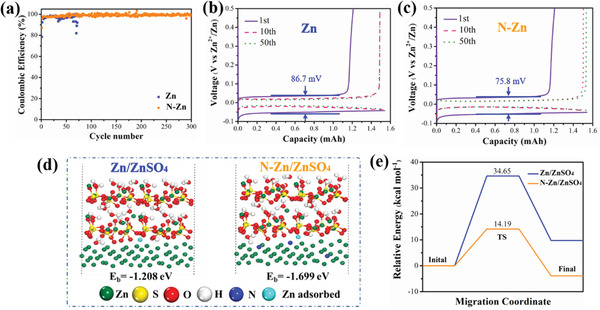
a) CE of Zn plating/stripping in Zn/CC and N‐Zn/CC cells. Typical GCD profiles of b) Zn/CC and c) N‐Zn/CC cells at 1 mA cm^−2^. d) Calculated binding energy of Zn^2+^ on Zn and N‐Zn electrodes. e) Zn ion migration barrier energy from Zn and N‐Zn electrodes to ZnSO_4_ electrolyte.

The Coulombic efficiency (CE), as one of the most important parameters for Zn metal electrodes, was tested against a carbon cloth (CC) electrode using a cutoff voltage of 0.5 V (versus Zn/Zn^2+^) at 1 mA cm^−2^ as shown in **Figure** **4**a. Typical galvanostatic charge/discharge (GCD) profiles of the bare Zn and N‐Zn electrodes are illustrated in Figure 4b,c, respectively. Initial CE of the bare Zn electrode was 78.97% with a polarization of 86.7 mV and the Zn/CC cell eventually died after around 70 cycles due to the formation of dendrites and byproducts on the Zn surface.^[^ ^37^ ^]^ In contrast, the N‐Zn electrode delivered an enhanced initial CE of 88.26% with a depressed polarization of 75.8 mV. In addition, the N‐Zn/CC cell maintained an average CE of 99.1% for over 300 cycles, confirming the good reversibility and fast Zn^2+^ kinetic of the N‐Zn electrode.^[^ ^38^ ^]^

To probe the N‐doping function on the Zn deposition process, Zn^2+^ adsorption and diffusion were investigated by density functional theoretical (DFT) computation as shown in Figure 4d. It is visible that the binding energy improved from −1.208 eV to −1.699 eV after N‐doping owing to the stronger interaction between Zn^2+^ and N atoms than Zn atoms. Thus, it can be deduced that the Zn^2+^ tends to first bond with the homogenized active sites of the N atoms on the Zn surface at the initial electrodeposition stage, leading to an even Zn plating process. Moreover, the Zn ion diffusion barriers from ZnSO_4_ electrolyte to the Zn and N‐Zn surface model were also calculated as illustrated in Figure S7, Supporting Information, and Figure 4e. The migration energy barrier of Zn to hydrated Zn ions on N‐Zn/ZnSO_4_‐electrolyte interface (14.19 kcal mol^–1^) was lower than that of Zn/ZnSO_4_ interface (34.65 kcal mol^–1^), which was in line with the reduced Zn nucleation potential for N‐Zn electrode. Hence, these results demonstrated that the plasma‐induced N‐doping not only facilitated Zn ions transportation but also enhanced interface impedance reduction.

**Figure 5:**
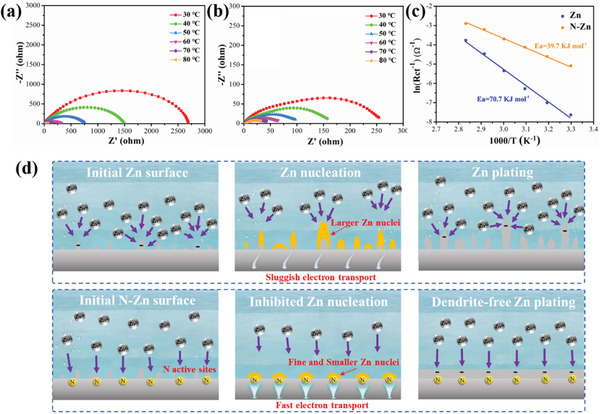
Nyquist plots of the a) Zn and b) N‐Zn symmetrical cells at different temperatures. c) Corresponding Arrhenius curves and comparison of activation energies of bare Zn and N‐Zn electrodes. d) Proposed mechanism for different Zn nucleation and plating behaviors of Zn and N‐Zn electrodes.

As the energy consumption for the de‐solvation of Zn^2+^ is commonly affirmed as the main barrier for the charge transfer migration, the activation energy (*E_a_*) value can be employed to represent the de‐solvation barrier of Zn^2+^ ions towards the electrode surface. To further explore the mechanism of deposition kinetics improvement of the N‐Zn electrode, the transfer and desolvation of Zn^2+^ were performed by the study of *E_a_* through the Arrhenius equation as follows:

(2) $$1/\;R_{ct}=\;Ae^{\left(-E_{a}/RT\right)}$$

where *R_ct_* is the charge transfer resistance at various temperatures, *A* is the frequency factor, *R* is the gas constant, and *T* is the absolute temperature. As shown in **Figure** **5**a,b, the *R_ct_* of N‐Zn was several orders of magnitude lower than that of bare Zn under different temperatures from 30 to 80 ℃. Moreover, the activation energy of N‐Zn was calculated as only 39.7 kJ mol^–1^ in contrast to that of bare Zn at 70.7 kJ mol^–1^ (Figure 5c), in agreement with the reduced migration energy barrier for N‐Zn as demonstrated by the theoretical simulation results. Therefore, it is evidenced that the expedited de‐solvation process of Zn ions also accounted for the superior kinetics of transfer for the N‐Zn electrode.

Based on the above analysis of electrochemical characters, different mechanisms for Zn nucleation and plating behaviors of Zn and N‐Zn electrodes can be proposed as illustrated in Figure 5d. Specifically, the doped N atoms on the N‐Zn electrode surface can work like Lewis base sites to interact with the Lewis acid anions (Zn^2+^) in the electrolyte.^[^ ^39^ ^]^ Regulated by the uniform N active sites, the Zn^2+^ ions are preferred to evenly distribute on the N‐Zn electrode, which thereby ensures the formation of fine and depressed Zn nuclei.^[^ ^40^ ^]^ Meanwhile, the rapid Zn desolvation property of the N‐Zn electrode can induce efficient electron transport and uniform distribution of Zn ions at the interface, finally resulting in an expected dendrite‐free Zn plating process. In contrast, the bare Zn foil surface experiences electron aggregation due to its initial rough morphology and sluggish electron transport speed, which in turn leads to severe dendrite formation.^[^ ^41^, ^42^ ^]^

**Figure 6:**
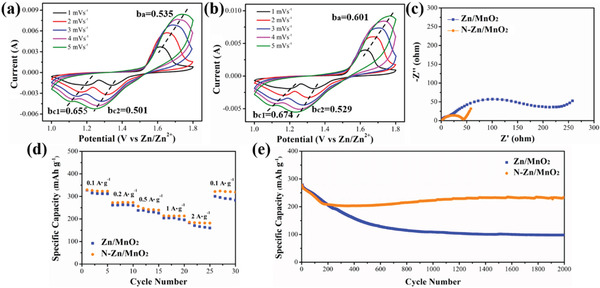
CV profiles of a) Zn/MnO_2_ and (b) N‐Zn/MnO_2_ cells under various scan rates from 1 mV s^–1^ to 5 mV s^–1^. c) Electrochemical impedance spectra, d) rate capacities, and e) long‐term cycling performance of Zn/MnO_2_ and N‐Zn/MnO_2_ cells at 1A g^–1^.

Furthermore, Zn/MnO_2_ cells with bare Zn or N‐Zn electrodes and aqueous electrolyte (2 m ZnSO_4_ and 0.2 m MnSO_4_) were assembled to test their practical performance. Specifically, the MnO_2_ cathode was synthesized by conducting a hydrothermal reaction as demonstrated in the literature and its crystal structure was indexed to *α*‐MnO_2_ by XRD pattern presented in Figure S8, Supporting Information.^[^ ^43^ ^]^ The Zn/MnO_2_ and N‐Zn/MnO_2_ cells were measured by cyclic voltammetry (CV) at 0.1 mV s^–1^ (Figure S9, Supporting Information). It is notable that N‐Zn/MnO_2_ cells delivered anodic peaks in lower potentials and cathodic peaks in higher potentials than bare Zn/MnO_2_ batteries. The largest potential position variation can impressively achieve 147 mV for their first cathodic peak. This suggested an accelerated charge/discharge kinetics of the full cell brought by the N‐Zn electrode, which is consistent with the tests of Zn/Zn and Zn/CC cells. Then the CV profiles of both cells were investigated under various higher scan rate from 1 to 5 mV s^–1^ as manifested in **Figure** **6**a,b. Similar to their profiles scanned at 0.1 mV s^–1^, both cells exhibited one anodic peak and two cathodic peaks at around 1.63V and 1.2/1.34V, respectively. Moreover, the CV profiles were used to distinguish different charge storage processes, as the peak current (*i*) and scan rate (*v*) obey the following equation:

(3) $$i\;=\;a\cdot v^{b}$$

where *a* and *b* are the adjustment parameters. The kinetic of ZIBs was mainly controlled by a capacitive process when the *b*‐value approaches 1, while a typical diffusion process dominates when the *b*‐value is close to 0.5. The *b* values for the anodic/cathodic peaks of Zn/MnO_2_ and N‐Zn/MnO_2_ cells were calculated as 0.535/0.655/0.501 and 0.601/0.674/0.529, respectively. The increased *b* values indicated that the N‐doped electrode interface facilitated the charge storage speed of the anode surface, thus resulting in an overall boosted electrochemical performance of the Zn/MnO_2_ cell.

The EIS measurements of the Zn/MnO_2_ and N‐Zn/MnO_2_ cells were subsequently conducted to study their kinetic differences, as illustrated in Figure 6c. Compared to the Zn/MnO_2_ cell, the interfacial impedance (*R_f_*) and charge‐transfer resistance (*R_ct_*) of the N‐Zn/MnO_2_ cell were significantly decreased, indicating an expedited Zn/charge transport speed on N‐doped electrode/electrolyte interface. In addition, the reduced charge transfer resistance of the N‐Zn electrode resulted in improved rate capability as shown in Figure 6d and S10, Supporting Information. Specifically, the N‐Zn electrode delivered discharge capacities of 329.1, 272.2, 256.2, 214.5, and 185.8 mAh g^–1^ at various current densities of 0.1, 0.2, 0.5, 1, and 2 A g^–1^, respectively. Furthermore, the capacity of the N‐Zn/MnO_2_ cell quickly recovered when the current density returned to 0.1 A g^–1^ at the 25th cycle (Figure S11, Supporting Information). In addition to rate capability, the outstanding surface performance of the N‐Zn electrode also led to enhanced cycling stability under 1 A g^–1^ (Figure 6e). Although the specific capacity of the Zn/MnO_2_ cell was slightly higher than that of the N‐Zn/MnO_2_ cell in the initial 30 cycles, the discharge capacity of the Zn/MnO_2_ cell declined sharply during the first 200 cycles resulted from the performance fading of the Zn electrode. In contrast, after the activation process for around 100 cycles, the capacity of the N‐Zn/MnO_2_ cell became stable and continuously increased due to the excellent electrochemical stability performance of the N‐Zn electrode. Such slow activation could be assigned to the sluggish desolvation of Zn ions on the MnO_2_ cathode/electrolyte interface.^[^ ^44^ ^]^ The phase transition process of MnO_2_ or the formation of reaction byproducts, such as Zn_4_SO_4_(OH)_6_·4H_2_O, may also result in the initial capacity fade of Zn/MnO_2_ cell as previously reported.^[^ ^45^ ^]^ Thus, it indicates the performance of the cathode has a huge impact on the capacity of the full battery when using excess Zn as anode. Furthermore, it is notable that the N‐Zn/MnO_2_ cell could maintain a high capacity of 230.9 mAh g^−1^ for 2000 cycles, indicating that the N‐doped Zn electrode surface effectively confined the dendrites as well as byproduct formation and hence led to remarkable cycling stability.

## 3 Conclusion

The nitrogen plasma was utilized to manipulate the interface electrochemistry of the Zn foil for developing an ultra‐stable anode for ZIBs. By virtue of the plasma treatment, uniform nitrogen doping on the surface of Zn foil was achieved, which led to the improved electrical conductivity and even more active sites for Zn nucleation. Due to these benefits, dendrite‐free Zn deposition ensured outstanding Zn plating and stripping performance with high average CE of 99.1% for 300 cycles. The symmetric N‐Zn cell demonstrated stable cycle performance for over 3000 h with a voltage hysteresis of 23 mV at 1 mA cm^–2^ (1 mAh cm^–2^). When assembled in a full ZIB with MnO_2_ cathode, a specific capacity of 230.9 mAh g^–1^ could be retained at a high current density of 1 A g^–1^ upon 2000 cycles. Therefore, the plasma‐induced N‐doping strategy for the surface treatment of Zn electrode opens up new avenues for the development of highly stable ZIB anodes with an ultra‐long lifespan and excellent electrochemical stability for industrial‐grade applications.

## Conflict of Interest

The authors declare no conflict of interest.

## Supporting information

Supporting Information

## Data Availability

Research data are not shared.
