# Supplementary material for: Advanced Zinc Anode with Nitrogen‐Doping Interface Induced by Plasma Surface Treatment
Source: Adv Sci (Weinh). 2021 Nov 26;9(3):2103952. doi: 10.1002/advs.202103952 (PMC8787405; doi:10.1002/advs.202103952)
Supplement: Supplementary file 1 — Supporting Information [file ADVS-9-2103952-s001.pdf]

ADVANCED SCIENCE
Open Access

Supporting Information

for *Adv. Sci.*, DOI: 10.1002/advs.202103952

Advanced Zinc Anode with Nitrogen-doping Interface induced by Plasma Surface Treatment

Hao Jia, Minghui Qiu, Chuntao Lan, Hongqi Liu, Mahmut Dirican, Shaohai Fu and Xiangwu Zhang**

Supporting Information

Advanced Zinc Anode with Nitrogen-doping Interface induced by Plasma Surface Treatment

Hao Jia[a], Minghui Qiu[a], Chuntao Lan[a], Hongqi Liu[a], Mahmut Dirican[b], Shaohai Fu[a,*] and Xiangwu Zhang[b,*]

[a] Key Laboratory of Eco-Textiles, Ministry of Education, Jiangnan University, Wuxi, Jiangsu, PR China.

[b] Fiber and Polymer Science Program, Department of Textile Engineering, Chemistry and Science, Wilson College of Textiles, North Carolina State University, Raleigh, NC 27695-8301, USA.

*Corresponding authors: Shaohai Fu, E-mail: shaohaifu@hotmail.com
Xiangwu Zhang, E-mail: xiangwu_zhang@ncsu.edu

Experimental Procedures

Preparation of N-Zn foils: The bare Zn foil was purchased from Beijing Saibo company (Beijing, China) and used without any treatment. The N-Zn foil was prepared by SY-DT03S type coupled with a high-frequency plasma generator. In a typical process, a bare Zn foil in thickness of 1 mm was cleaned with acetone, absolute ethyl alcohol and deionized water, and subsequently dried in a vacuum oven. After cleaning, the bare Zn foil was treated on the glow discharge reactor to obtain the N-Zn foil at output power of 200 W in N_2 atmosphere for 2 min. Finally, the N-Zn foil was cut into ϕ 14 mm round pieces for further tests.

Fabrication of α-MnO_2: In a typical synthesis process, 3 mmol $MnSO_4 \cdot H_2O$ and 2 mL 0.5 mol L^{-1} H_2SO_4 were dissolved in 60 mL distilled water under magnetic stirring for 30 min and then 20 mL 0.1 mol·L^{-1} $KMnO_4$ was slowly added into the above solution. The mixture was stirred for 1 h, followed by sonication for 1 h. Finally, the mixture

was transferred into a Teflon-lined autoclave and heated at 120 ℃ for 12 h. After cooling to room temperature, α-MnO_2 was washed with distilled water and freeze-dried.

Preparation of MnO_2 cathode. The MnO_2 cathode was prepared by a blading coating method. Briefly, MnO_2 powder, black carbon and PVDF with a weight ratio of 8:1:1, were homogeneously mixed in N-methyl pyrrolidone (NMP) under continuous ultrasonic stirring. Then the slurry was uniformly coated on the surface of stainless-steel collector and dried in a vacuum at 40 ℃ for 12 h. Finally, the as-prepared electrodes were cut into round pieces of Φ 14 mm in average mass loading of approximate 2 $mg·cm^{-2}$.

Assembly of Zn/Zn Symmetric Cells and Zn/MnO_2 Cells: CR2032 coin-type Zn/Zn symmetric cells were assembled with identical electrodes of bare Zn or N-Zn, 2 M $ZnSO_4$ electrolyte and glass fiber separator (Whatman GF/D). For the Zn/MnO_2 batteries, MnO_2 cathode and bare Zn (or N-Zn) were separated by glass fiber separator. A mixture of 2 M $ZnSO_4$ and 0.2 M $MnSO_4$ was used as the electrolyte.

Materials Characterization: The morphologies of bare Zn and N-Zn were characterized by Field emission scanning electron microscope (FSEM, ZEISS-SIGMA HD) equipped with energy-dispersive spectroscopy (EDS, TEAM Octane EDS-30, AMETEK Co.) at an accelerating voltage of 15 kV for elemental analysis. The electron probe X-ray microanalyzer (EPMA, JXA-8530F PLUS) was also conducted to confirm the uniform nitrogen distribution on the treated Zn surface. The morphologies of bare Zn and N-Zn in symmetric cells during Zn plating process were observed by the optical microscope (DM2007P, Wetzlar, Germany), where the Zn electrodes (length: 20 mm, width: 5 mm, and thickness: 0.1 mm) were fixed onto a transparent glass plate to construct a homemade optical cell. The backs of the Zn electrodes were covered with a coat of packaging tape. The surface wettability of Zn

electrodes was performed by a contact angle measuring system (JC2000D1, Powereach, China). The phase and structure of bare Zn and N-Zn were further identified by X-ray diffraction (XRD, Rigaku Ultima IV) with Cu Kα radiation (λ = 0.15418 nm). XPS (ESCALAB 250XI, Thermo Scientific, USA) was used to characterize the element type and atomic configurations of bare Zn and N-Zn. Ultra-depth three-dimensional microscope (VHX-1000C, Keyence, Japan) was carried out to observe the micromorphology of the bare Zn and N-Zn electrode before and after cycling test.

Electrochemical Measurement: The Zn electrodeposition was monitored in a transparent symmetric cell with an in situ optical microscope (DM2007P, Wetzlar, Germany). Coulombic efficiency (CE) test was performed against CC electrode by plating 1 mAh·cm^{-2} of Zn and then stripping to 0.5 V. Cyclic voltammetry (CV) curves were conducted on an electrochemical workstation (CHI 660E) with a voltage window of 1.0 to 1.8 V at different scan rates. The galvanostatic charge/discharge (GCD) measurements were performed by a battery test system (LAND, CT3001A) at different current densities. Electrochemical impedance spectra (EIS) were obtained on an electrochemical workstation (CHI 660E) in a frequency range from 100 kHz to 0.1 Hz.

DFT Calculation: All spin-polarization density functional theory (DFT) calculations were performed by employing the first-principles within the generalized gradient approximation (GGA) using the Perdew-Burke-Ernzerhof (PBE) formulation.[1-2] The projected augmented wave (PAW) potentials were selected to describe the ionic cores and valence electrons were taken into account using a plane wave basis set with a kinetic energy cutoff of 400 eV.[3-4] Partial occupancies of the Kohn-Sham orbitals were allowed using the Gaussian smearing method and a width of 0.05 eV. The electronic energy was considered self-consistent when the energy change was smaller than 10^{-6} eV. A geometry optimization was considered convergent when the energy

change was smaller than 0.05 eV $Å^{-1}$. The vacuum spacing in a direction perpendicular to the plane of the structure was 15 Å. The Brillouin zone integration was performed using 2×2×1 Monkhorst-Pack *k*-point sampling. Finally, the Binding energies (Eb) were calculated as $E_b = E_{Zn/sub} - E_{Zn} - E_{sub}$, where $E_{Zn/sub}$, E_{Zn}, and E_{sub} were the total energies of the optimized adsorbate/substrate system, the Zn atom in the structure, and the clean substrate, respectively. Zn ions migration barrier energies were also evaluated using the climbing nudged elastic band (CI-NEB) methods.

References

[1] G. Kresse, J. Furthmüller, *Comput. Mater. Sci.* **1996**, *6*, 15.

[2] J.P. Perdew, K. Burke, M. Ernzerhof, *Phys. Rev. Lett.* **1996**, *77*, 3865.

[3] G. Kresse, D. Joubert, *Phys. Rev. B* **1999**, *59*, 1758.

[4] P.E. Blöchl, *Phys. Rev. B* **1994**, *50*, 17953.

Table S1. N doping content of each sample under different treating time.

Treating Time (min)	N doping content
1	3.6%
2	6.1%
3	5.1%
4	4.9%

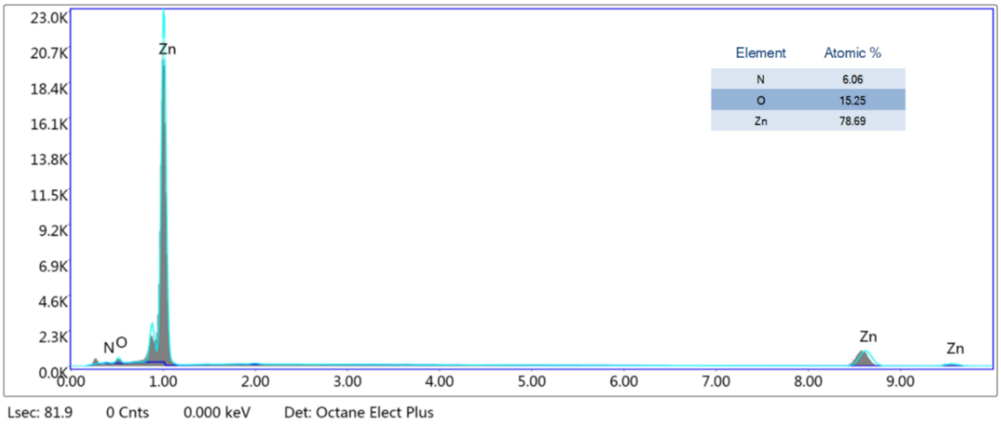

Figure S1. EDS pattern of N-Zn foil.

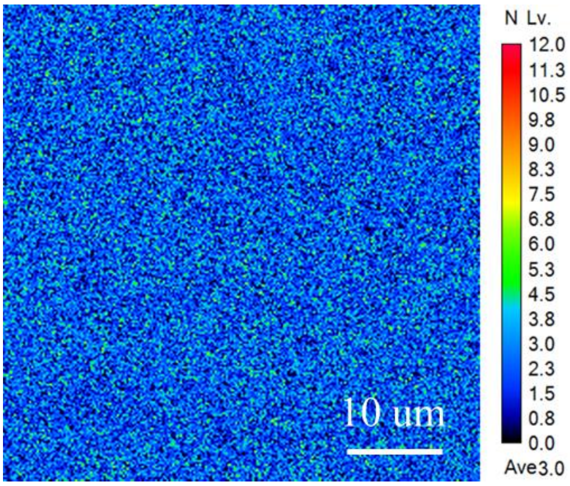

Figure S2. EPMA image of N-Zn.

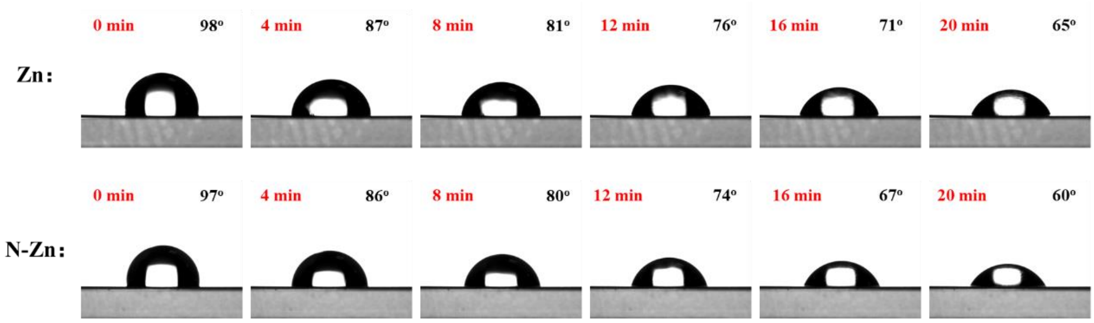

Figure S3. Images of contact angles of the 2 M $ZnSO_4$ electrolyte on Zn and N-Zn electrodes during a 20 min period.

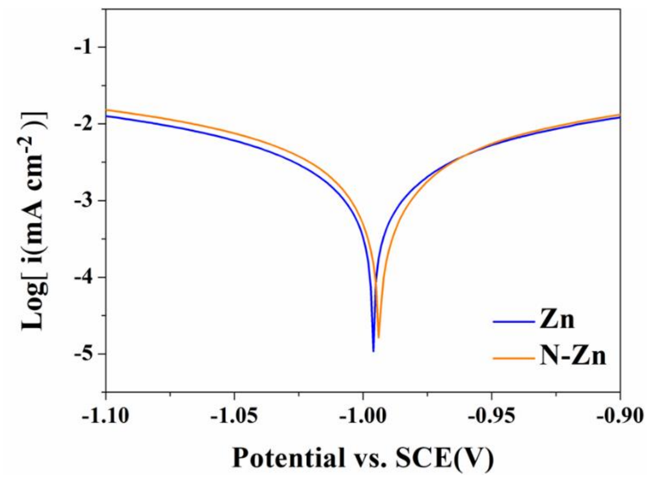

Figure S4. Linear polarization curves of the corrosion on bare Zn and N-Zn electrodes.

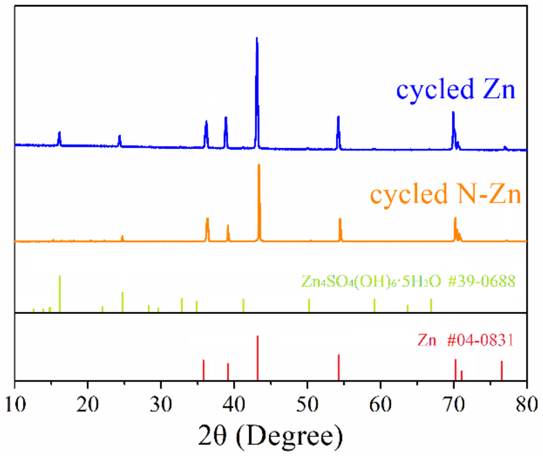

Figure S5. XRD patterns of Zn and N-Zn electrodes after 50 cycles under the current density of 1 mA·cm^{-2}.

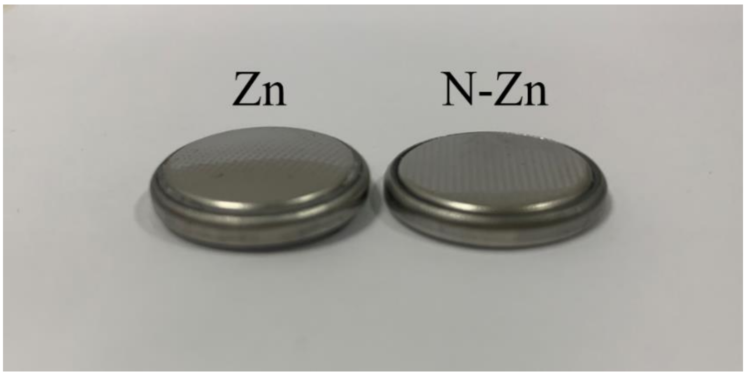

Figure S6. Image of cycled symmetric Zn and N-Zn cells.

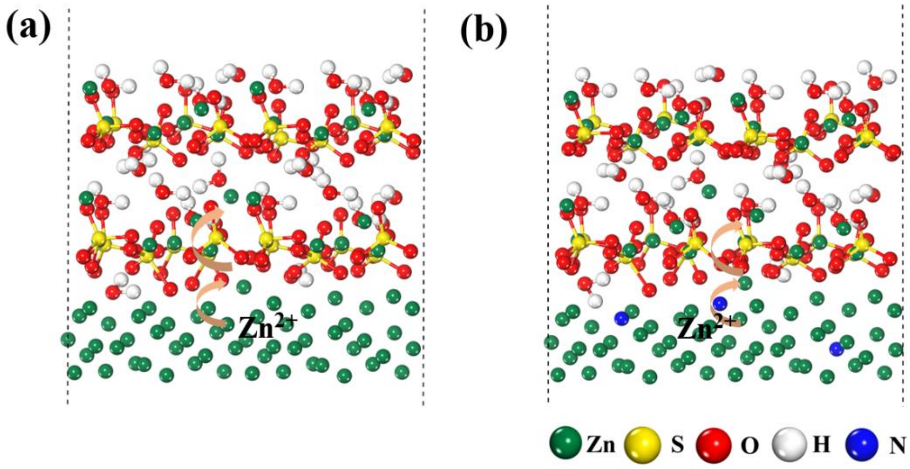

Figure S7. Diffusion paths of a Zn ion from (a) Zn and (b) N-Zn electrodes to $ZnSO_4$ electrolyte.

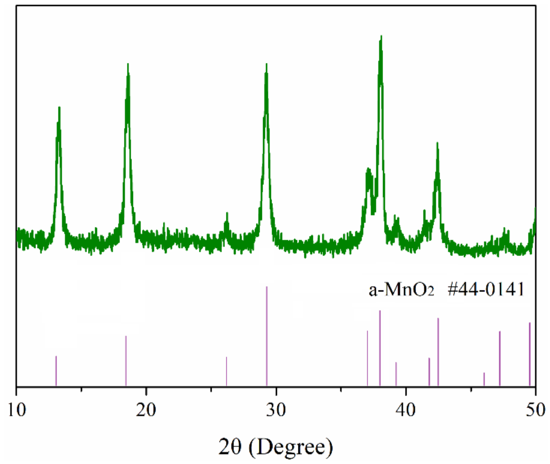

Figure S8. XRD pattern of α-MnO_2.

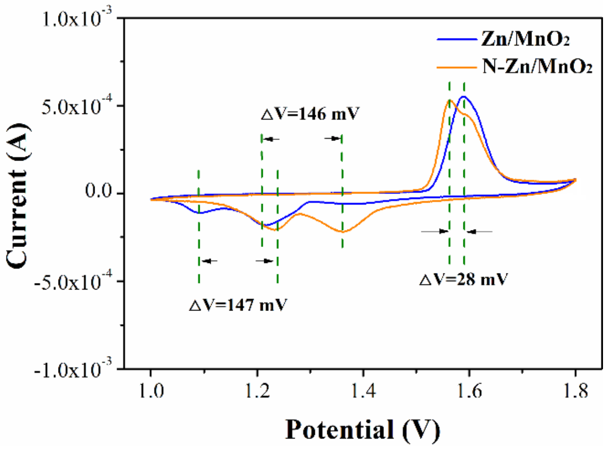

Figure S9. CV profiles of Zn/MnO_2 and (b) N-Zn/MnO_2 cells under 0.1 $mV{\cdot}s^{-1}$.

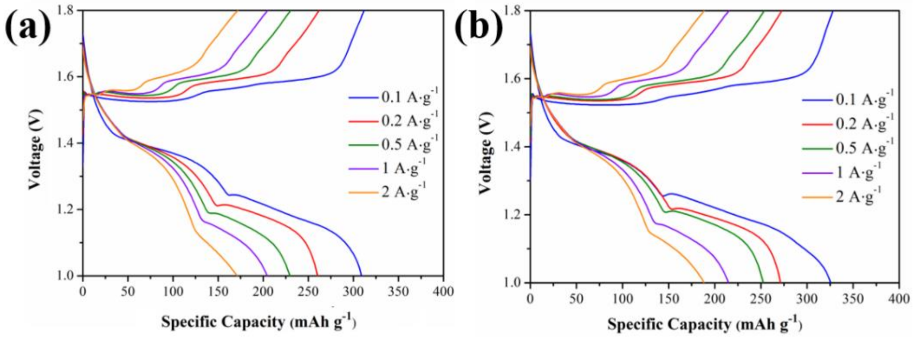

Figure S10. Charge/discharge profiles of (a) Zn/MnO_2 and (b) N-Zn/MnO_2 cells under various densities from 0.1 $A{\cdot}g^{-1}$ to 2 $A{\cdot}g^{-1}$.

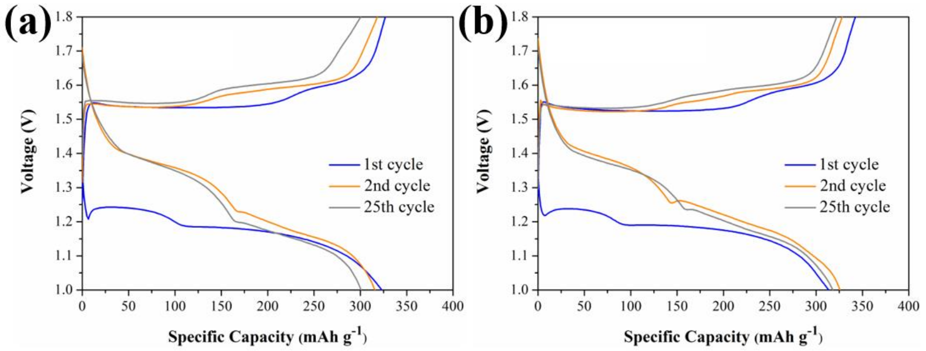

Figure S11. Charge/discharge profiles of (a) Zn/MnO_2 and (b) N-Zn/MnO_2 cells at the specific cycle.
